# Kidney Cyst Lining Epithelial Cells Are Resistant to Low-Dose Cisplatin-Induced DNA Damage in a Preclinical Model of Autosomal Dominant Polycystic Kidney Disease

**DOI:** 10.3390/ijms232012547

**Published:** 2022-10-19

**Authors:** Sayanthooran Saravanabavan, Gopala K. Rangan

**Affiliations:** 1 Michael Stern Laboratory for Polycystic Kidney Disease, Westmead Institute for Medical Research, The University of Sydney, Westmead, NSW 2145, Australia; 2 Department of Renal Medicine, Westmead Hospital, Westmead, NSW 2145, Australia

**Keywords:** ataxia telangiectasia mutated, DNA damage response signaling, γH2AX

## Abstract

Increased DNA damage response (DDR) signaling in kidney cyst-lining epithelial cells (CECs) may provide an opportunity for cell-specific therapeutic targeting in autosomal dominant polycystic kidney disease (ADPKD). We hypothesized that inhibiting ataxia telangiectasia mutated (ATM; a proximal DDR kinase) together with low-dose cisplatin overwhelms the DDR response and leads to selective apoptosis of cyst-lining epithelial cells (CECs). *Pkd1^RC/RC^/Atm^+/−^* mice were treated with either vehicle or a single low-dose cisplatin, and the acute effects on CECs (DNA damage and apoptosis) after 72 h and chronic effects on progression (cyst size, inflammation, fibrosis) after 3 weeks were investigated. At 72 h, cisplatin caused a dose-dependent increase in γH2AX-positive nuclei in both CECs and non-cystic tubules but did not cause selective apoptosis in *Pkd1^RC/RC^/Atm^+/−^* mice. Moreover, the increase in γH2AX-positive nuclei was 1.7-fold lower in CECs compared to non-cystic epithelial cells (*p* < 0.05). Low-dose cisplatin also did not alter long-term disease progression in *Pkd1^RC/RC^/Atm^+/−^* mice. In vitro, human ADPKD cyst-derived cell lines were also resistant to cisplatin (WT9-12: 61.7 ± 4.6%; WT9-7: 64.8 ± 2.7% cell viability) compared to HK-2 (25.1 ± 4.2%), and 3D cyst growth in MDCK cells was not altered. Finally, combined low-dose cisplatin with AZD0156 (an ATM inhibitor) non-selectively reduced γH2AX in both cystic and non-cystic tubular cells and exacerbated cystic kidney disease. In conclusion, these data suggest that CECs are resistant to DNA damage, and that the combination of cisplatin with ATM inhibitors is not an effective strategy for selectively eliminating kidney cysts in ADPKD.

## 1. Introduction

Autosomal dominant polycystic kidney disease (ADPKD) affects approximately 12 million people worldwide and is mostly due to inherited mutations in either PKD1 or PKD2 genes [1]. The disease is characterized by the slow growth of multiple focal fluid-filled kidney cysts which leads to a progressive decline in renal function starting in early adulthood and culminating in kidney failure by middle age [2]. Tolvaptan (a selective vasopressin receptor antagonist) is currently the only specific disease-modifying drug approved for use in ADPKD. It slows the decline of kidney function (glomerular filtration rate) by approximately 1 mL/min/1.73 m^2^/year but is limited by side effects of polyuria and hepatotoxicity, and thus other disease-modifying drugs need to be developed [3,4].

The molecular mechanisms of kidney cyst formation share common signal transduction pathways to neoplastic transformations [5]. For example, increased proliferation, loss of cellular differentiation, and genomic instability and mutation are pathological features in ADPKD common to many cancers [6,7]. In a previous study, we demonstrated that ataxia telangiectasia mutated (ATM), a key sensor of DNA double-strand breaks (DSBs) was upregulated in human CECs, and in in vitro and in vivo models of ADPKD [6]. In human ADPKD cells, increased ATM expression was associated with survival following exogenous H_2_O_2_ treatment [6]. Genetic inhibition of ATM (*Atm^+/−^* or *Atm^−/−^*) did not however alter cystic disease in *Pkd1^RC/RC^* mice [8] and although the small-molecule ATM inhibitor, AZD0156, reduced proliferation and increased p53 in *Pkd1^RC/RC^* kidneys, it did not induce selective apoptosis of CECs [8].

The survival benefit of cancerous cells has been successfully targeted by combining DDR kinase inhibitors with cytostatic agents such as cisplatin to cause DSB accumulation, mitotic catastrophe and apoptosis [9,10]. Cisplatin forms platinum adducts by intra- and inter-strand cross linking of purine bases (guanine, adenine) in DNA [11], which in cancer cells with aberrant DNA replication and dysregulated DDR, leads to DSB accumulation, mitotic catastrophe and apoptosis [9]. In previous studies, cisplatin treatment sensitized ATM-deficient non-small cell lung cancer cells to radiation [12] and PTEN-deficient breast cancer cells to ATM kinase inhibition [13]. Of the many cytostatic agents used in cancer, cisplatin has been extensively studied as a nephrotoxic agent, and in preclinical murine models has demonstrated acute kidney injury at high doses (>10 mg/kg) and transformation to chronic kidney disease at repeated low doses [14,15,16,17]. A single low dose of cisplatin however induced DNA damage in tubular epithelia without causing acute injury or transformation to chronic kidney disease [17].

In this study we tested the hypothesis that the combination of ATM inhibition and low-dose cisplatin causes the targeted death of CECs in ADPKD without affecting non-cystic tissue compared to ATM inhibition alone. The specific aims of the study were to determine the: (i) acute effects of low-dose cisplatin on DNA damage formation and apoptosis in CECs and normal tubules in *Pkd1^RC/RC^/Atm^+/−^*; (ii) chronic effects of low-dose cisplatin on cystic disease progression of *Pkd1^RC/RC^/Atm^+/−^* mice following 3-weeks and (iii) in vitro effects of cisplatin on the survival of cell lines derived from human ADPKD (compared to normal kidney tubular cells) and on MDCK cyst growth; and finally (iv) acute effects of low-dose cisplatin with AZD0156 on DNA damage formation and apoptosis in CECs and normal tubules in *Pkd1^RC/RC^* mice.

## 2. Results

### 2.1. Cyst Lining Epithelial Cells of Pkd1^RC/RC^/Atm^+/−^ Mice Are Resistant to Low-Dose Cisplatin

**Figure 1:**
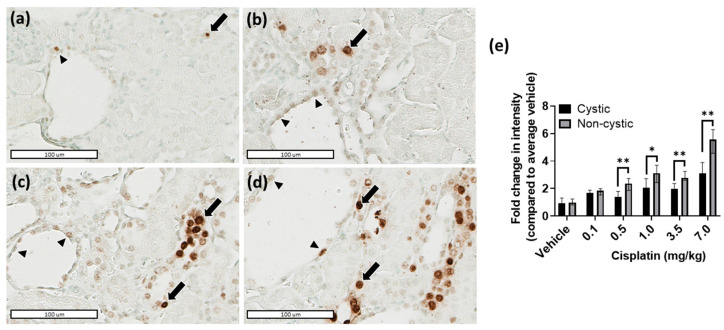
Cyst lining epithelia are resistant to cisplatin-induced DNA damage. Immunohistochemical staining for γH2AX in kidney cortical sections of *Pkd1^RC/RC^/Atm^+/−^* mice treated with (**a**) vehicle, (**b**) 1.0 mg/kg cisplatin, (**c**) 3.5 mg/kg cisplatin and (**d**) 7.0 mg/kg cisplatin, demonstrating increased staining intensity of non-cystic tubules (arrows) compared to cyst lining epithelia (arrowheads); (**e**) Histogram showing fold change of γH2AX intensity from baseline (vehicle) in cyst lining epithelia and non-cystic tubules of *Pkd1^RC/RC^/Atm^+/−^* kidneys. Graphs showing median values with error bars representing upper and lower quartiles. Scale bar = 100 µm. * *p* < 0.01; ** *p* < 0.001.

In *Pkd1^RC/RC^/Atm^+/−^* mice, cisplatin caused a dose-dependent increase in γH2AX positive nuclei and kidney tubular injury (Figure 1; Figure S1). Unexpectedly, the intensity of γH2AX in CECs was lower compared to non-cystic tubules, with the difference increasing at higher doses of cisplatin (Figure 1). At 7.0 mg/kg of cisplatin, the increase in γH2AX positive nuclei was 3.14 ± 1.18-fold in CECs and 5.43 ± 0.94-fold in non-cystic epithelial cells compared to vehicle (*p* < 0.05) (Figure 1e).

**Figure 2:**
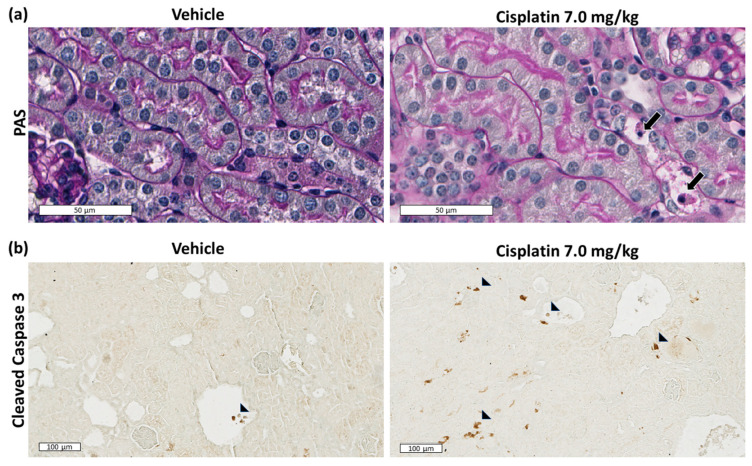
Apoptosis in cisplatin treated *Pkd1^RC/RC^/Atm^+/−^* mice. (**a**) Periodic acid Schiff (PAS) staining of cortical kidney sections showing apoptotic cells in non-cystic tubules (arrows) in cisplatin treated mice (Scale bars = 50 µm); (**b**) Immunohistochemical staining for cleaved caspase 3 showing positive nuclei (arrowheads) that are increased focally in non-cystic tubules of cisplatin treated mice (Scale bars = 100 µm).

By light microscopy, increased tubular damage was observed with higher dose of cisplatin, and at 3.5 and 7.0 mg/kg cisplatin, increased apoptotic cells were observed in the non-cystic tubules. This was confirmed by immunohistochemistry for the apoptosis marker, cleaved caspase-3 which showed focally positive nuclei that was increased in non-cystic tubules but not altered in CECs compared to vehicle treatment (Figure 2).

The resistance of CECs to low-dose cisplatin compared to non-cystic epithelial cells was also observed in kidneys of *Pkd1^RC/RC^* mice (normal ATM) in a separate study from our laboratory (unpublished).

### 2.2. Chronic Effects of Low-Dose Cisplatin on Cystic Kidney Disease in Pkd1^RC/RC^/Atm^+/−^ Mice

**Figure 3:**
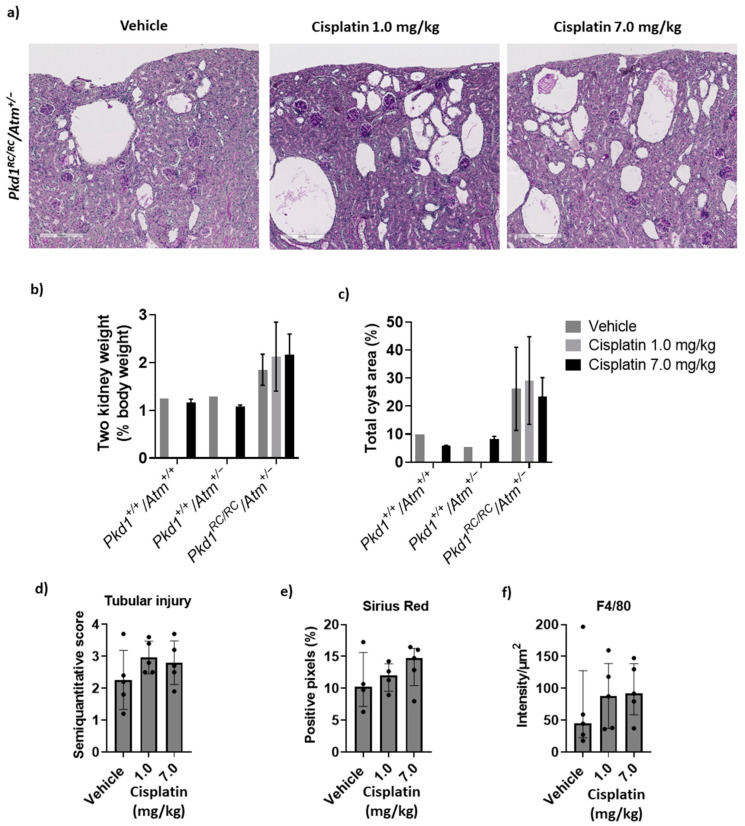
Long-term effects of cisplatin on tubular injury and cyst growth. (**a**) Morphology of outer cortical region of mice treated with vehicle, cisplatin 1.0 mg/kg or cisplatin 7.0 mg/kg; (**b**) two-kidney weight as a percentage of body weight at sacrifice; (**c**) cyst area in outer cortical region (scale bars = 200 µm); (**d**) semiquantitative score of tubular injury; (**e**) quantification of collagen deposition by Sirius red staining showing strong positive pixels per µm^2^; (**f**) quantification of F4/80 immunostaining showing strong positive pixels per µm^2^. Graphs showing median values with error bars representing upper and lower quartiles.

As low doses of cisplatin were used, treatment with either 1.0 mg/kg or 7.0 mg/kg did not lead to any chronic changes in cyst area, inflammation, or fibrosis after 3 weeks. Markers of chronic cystic progression, such as kidney enlargement and percentage cystic area was not different between groups (Figure 3). Furthermore, the progression of interstitial myofibroblast and monocyte accumulation and collagen deposition were not altered by cisplatin in *Pkd1^RC/RC^/Atm^+/−^* mice (Figure 3; Figures S2–S5).

### 2.3. In Vitro Effects of Cisplatin on Human ADPKD Cells and 3D-MDCK Cyst Growth

**Figure 4:**
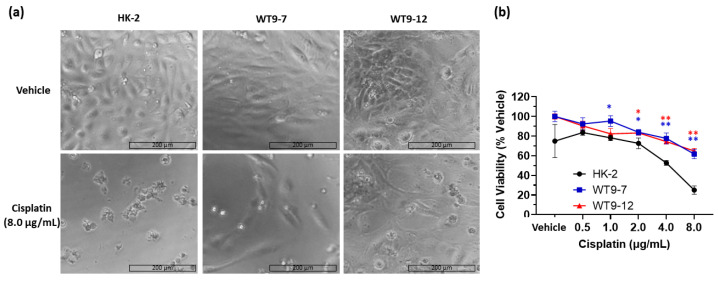
Effect of cisplatin treatment on human ADPKD cells (WT9-7 and WT9-12) compared to control (HK-2) cells. (**a**) Cell morphology under light microscope showing decreased cell density and necrosed HK-2 cells compared to WT9-7 and WT9-12 cell lines with 8.0 µg/mL cisplatin treatment; (**b**) Quantitative measurement of cell viability using the MTT assay showing significantly reduced cell viability in HK-2 cells compared to WT9-7 and WT9-12 cells at similar doses of cisplatin. * *p* < 0.05; ** *p* < 0.01 compared to HK-2 cells at respective doses with the color of asterisk matching line color. Scale bar = 200 µm.

Consistent with the in vivo findings in *Pkd1^RC/RC^/Atm^+/−^* mice, ADPKD cell lines were also resistant to death in response to cisplatin compared to normal human kidney cells (HK-2) (Figure 2a). As measured by the MTT assay, treatment with 8 µg/mL cisplatin reduced the viability of HK-2 cells to 25.1 ± 4.2%, whereas in WT9-7 and WT9-12 cells it was 61.7 ± 4.6% and 64.8 ± 2.7%, respectively, demonstrating ~37–40% increased survival in the latter (Figure 4).

**Figure 5:**
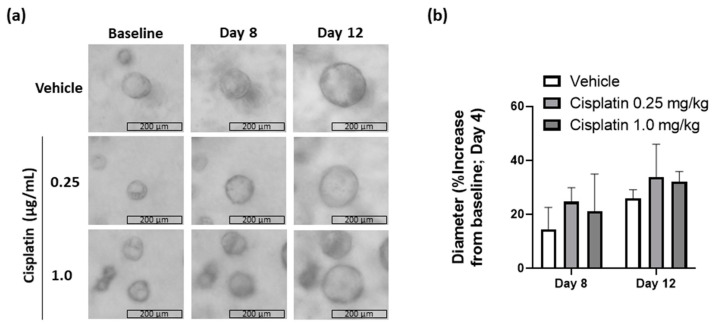
Long-term effects of cisplatin on cyst growth. (**a**) Representative images of individual cysts treated with vehicle or cisplatin at respective doses on Day 4 (baseline) and growth observed on Day 8 and Day 12; (**b**) quantification of cyst diameter from images acquired on Day 8 and Day 12 and normalized to diameter at baseline (Day 4). Graphs showing median values with error bars representing upper quartiles. Scale bar = 200 µm.

The effect of low-dose cisplatin on long-term cyst progression was confirmed in MDCK cysts. Non-toxic doses were determined as 0.25 and 1.0 µg/mL using the MTT assay. At these doses, cyst growth with cisplatin treatment was not different to vehicle treatment up to 12 days follow up (Figure 5).

### 2.4. AZD0156 in Combination with Low-Dose Cisplatin Non-Specifically Reduces γH2AX

**Figure 6:**
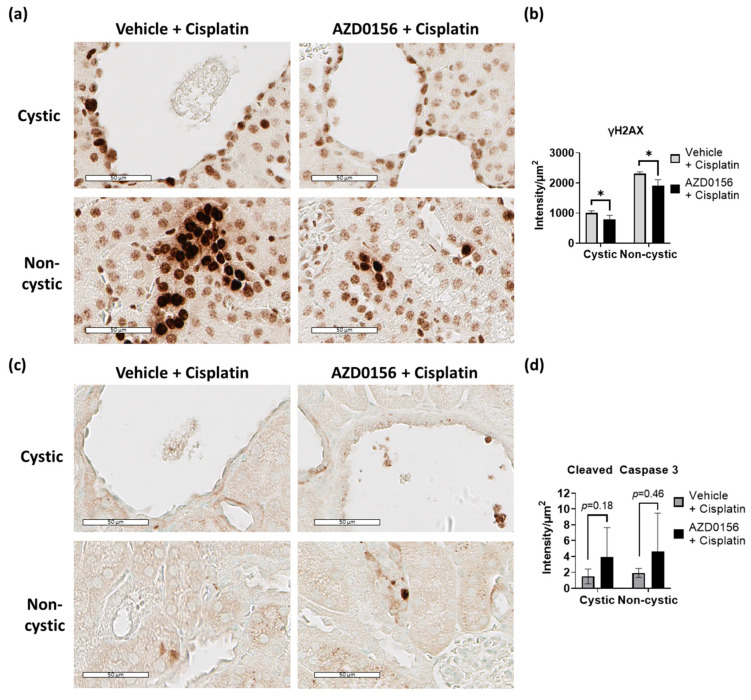
Pharmacological inhibition of ATM using AZD0156 in combination with low-dose cisplatin. (**a**) Immunohistochemical staining for γH2AX and (**b**) quantification of intensity of strong positive γH2AX pixels in cystic and non-cystic kidney tubules of mice treated with cisplatin combined with AZD0156 or vehicle (*n* = 4–5 per group); (**c**) Immunohistochemical staining for cleaved caspase-3 and (**d**) quantification of intensity of strong positive pixels in cystic and non-cystic kidney tubules of mice treated with cisplatin combined with AZD0156 or vehicle (*n* = 4–5 per group). Graphs showing mean with error bars representing standard deviation; * *p* < 0.05. Scale bars = 50 µm.

Because heterozygosity for ATM only causes a partial reduction of protein function, additional experiments were performed to evaluate the effects of AZD0156 in combination with low-dose cisplatin on cystic kidney injury and disease in *Pkd1^RC/RC^* mice. As shown in Figure 6, treatment with AZD0156 (20 mg/kg) reduced the number and intensity of γH2AX positive pixels at 72 h following i.p. cisplatin (7.0 mg/kg). The reduction in γH2AX was observed in both CECs and normal tubules with near complete reduction in two of the four mice treated (Figure 6a,b). Furthermore, staining for apoptosis marker, cleaved caspase 3 was focal in nuclei of CECs and normal tubules, and in cytosol and periphery of dilated tubules and cysts. Although there was a trend towards an increase in apoptosis following treatment with AZD0156, there was variability within groups in the number of strong positive staining per cyst and differences did not reach statistical significance (*p* = 0.18 in CECs and *p* = 0.46 in non-cystic tissue) (Figure 6c,d).

**Figure 7:**
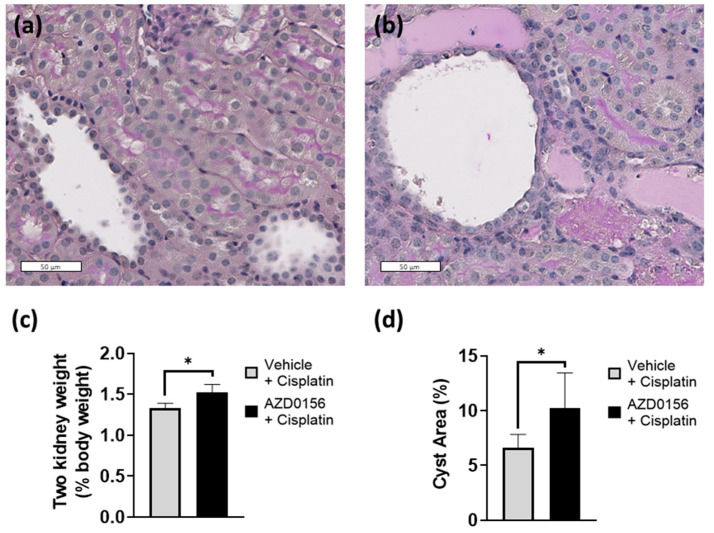
Pharmacological inhibition of ATM using AZD0156 increases tubular damage and cyst growth by 72 h in *Pkd1^RC/RC^* mice. Morphology of outer cortical region of mice treated with cisplatin combined with (**a**) vehicle or (**b**) AZD0156. Histograms showing (**c**) two-kidney weight as a percentage of body weight at sacrifice and (**d**) percentage cyst area in outer cortical region (*n* = 4–5 per group). Graphs showing mean with error bars representing standard deviation; * *p* < 0.05. Scale bars = 50 µm.

Morphological assessment was consistent with non-specific damage and showed exacerbated cystic kidney disease with AZD0156 and cisplatin combination (Figure 7).

## 3. Discussion

In this study we investigated for the first time whether the combination of low-dose cisplatin together with ATM inhibition could selectively sensitize and induce mitotic catastrophe in CECs. Contrary to this hypothesis, we found that CECs exhibited less γH2AX formation and apoptosis compared to non-cystic tubular epithelial cells in response to a single low dose cisplatin, without affecting long-term disease progression. These findings were corroborated by in vitro studies demonstrating that human ADPKD cell lines were resistant to cisplatin compared to normal human kidney tubular cell lines. Furthermore, neither genetic inhibition of ATM (*Atm^+/−^*) nor pharmacological inhibition using small molecule ATM inhibitor (AZD0156) in *Pkd1^RC/RC^* mice selectively altered the viability of CECs. Taken together, these findings suggest that CECs have an increased capacity to survive under exogenous DNA damage and this is not dependent on ATM.

In our previous studies, increased ATM in vitro ADPKD cell lines (WT9-7 and WT9-12 cells) was associated with resistance to exogenous H_2_O_2_ mediated injury [8] which indicated a possible role for ATM in the survival benefit of CECs. The primary function of ATM is to sense DNA DSBs and activate the G1/S and G2 checkpoints in the cell cycle to facilitate repair prior to entering mitosis [18]. RNA-seq studies in wildtype mice demonstrate that the ATM signaling pathway is one of the most up-regulated in kidneys in response to cisplatin [19]. In cisplatin treated murine leukemia (L120/0) cells, it was found that DSBs were the earliest sign of cells that escaped the G2 checkpoint and eventually underwent cell death [18]. Increased DNA DSBs and accelerated kidney injury was thus observed in mice administered cisplatin combined with small molecule ATM inhibitors [20]. Given the role of PKD1 in cell cycle checkpoint control [21], and low PKD1 resulting in increased ATM [8], we expected ATM inhibition to cause DSB accumulation and mitotic catastrophe of CECs. The increased apoptosis that we observed in the cystic and non-cystic tubules of *Pkd1^RC/RC^/Atm^+/−^* mice in response to cisplatin are possibly explained by the above mechanisms. In CECs of *Pkd1^RC/RC^/Atm^+/−^* mice however, fewer γH2AX foci indicates that DNA damage is efficiently repaired despite reduced ATM, and that alternate mechanisms may underlie the survival benefit in CECs. Furthermore, pharmacological inhibition of ATM using AZD0156 reduced γH2AX foci and exacerbated cystic kidney disease. The additional adverse effects on kidney injury with AZD0156 compared to *Pkd1^RC/RC^/Atm^+/−^* mice are likely due to differences in mechanisms of ATM suppression with the two methods. Pharmacological inhibition reduces ATM signaling and the binding of alternate kinases such as ATR, which may otherwise be recruited in the genetically inhibited ATM model [22]. The importance of the upstream DDR kinases in preventing maladaptive repair has been demonstrated [23] and may thus explain the rapid cyst growth observed herein.

While the role of DNA damage in ADPKD is still not completely understood, parallels can be derived from cancer where the effect of cisplatin on DDR signaling has been extensively studied [24,25,26]. In human urinary bladder cancer, similar to our observations in this study, increased γH2AX staining was observed in early superficial and early invasive lesions and reduced in more advanced primary carcinomas [24]. The underlying mechanisms may however be different; while the advanced carcinomas suppressed the DDR and proceeded through the cell cycle [24], our previous studies showed increased activation of DDR [6] suggesting more efficient clearing of DNA damage. An intrinsic resistance to cisplatin is also seen in patients with colorectal, prostate, lung and breast cancers [24,25,26,27] and survival despite platinum-DNA adduct formation has been associated with enhanced clearing by the nucleotide excision repair (NER) pathway, or by tolerance mechanisms such as translesion synthesis [28]. Up regulation of these DDR pathways in CECs was demonstrated in our previous study [6], however requires further testing.

Single low dose cisplatin caused DNA damage in both cystic and non-cystic kidney tubules, and in *Pkd1^RC/RC^/Atm^+/−^* mice did not induce chronic changes in cyst growth, fibrosis or inflammation and therefore remains a potential strategy to induce DSBs in CECs when specific mechanisms promoting survival are identified. Cisplatin was specifically chosen as adjunctive agent because of the substantial preclinical evidence for its effect in the kidney and its ease of access, as well as previous studies in cancer in combination with ATM inhibition [10]. Other cytostatic agents such as alkylating agents and taxanes have also shown nephrotoxicity and wide systemic toxicity [29,30] but have not been as extensively studied. Although ADPKD presents increased susceptibility to kidney injury [31,32,33], the low dose of cisplatin tested in this study caused DNA damage without chronic injury. The DNA damage and apoptosis observed at 72 h in the non-cystic tubules resolved by three weeks without causing changes in cyst area, chronic inflammation, or fibrosis. This is in line with studies showing that single low doses of cisplatin (≤7 mg/kg) do not decrease renal function (BUN and serum creatinine) or increase chronic inflammation and fibrosis [15].

The strengths of our study include the use of a double-mutated mouse model; the *Pkd1^RC/RC^* hypomorphic mutation that resembles slow disease in humans [34], together with a truncation mutation of the ATM gene [35]. Demonstrated low dose cisplatin [15,17] in conjunction with the double-mutated model proved ideal for causing DSBs without progression to chronic injury in the non-cystic tubules. The resistance of human ADPKD cells in vitro to cisplatin-induced DNA damage further strengthens the primary results and validates the findings. There are however possible limitations of the study; *Atm^+/−^* mice have checkpoint inactivation levels intermediate between *Atm^+/+^* and *Atm^−/−^* [36] and therefore a certain level of ATM activity may still be present in CECs of *Pkd1^RC/RC^/Atm^+/−^* mice. Although *Atm^−/−^* genotype would have led to complete suppression of ATM, it is accompanied by developmental defects and heightened sensitivity to DNA DSBs [35]. Furthermore, complete inhibition of a critical DDR kinase would not be reflective of a translational approach in a slowly progressing disease such as ADPKD. To confirm our findings, we inhibited ATM pharmacologically [37] in the *Pkd1^RC/RC^* mice using a high dose of ATM inhibitor (20 mg/kg) which causes complete reduction of chemoresistance from ATM activity [38]. The level of ATM protein could not however be determined due to a lack of suitable cross-reacting antibodies and increased background staining [6]. The non-specific increase in apoptosis with cisplatin in both our models of ATM inhibition adds strength to our conclusion of ATM not having a specific role in CECs. The effect of lower doses of AZD0156 on tubular damage and selectivity however may be different and was not tested in this study.

In conclusion, ATM inhibition together with low-dose cisplatin did not specifically sensitize CECs to DNA damage and apoptosis. These data suggest that CECs are not specifically dependent on ATM for survival even under genotoxic stress conditions and therefore combination of DNA damaging chemotherapeutics with ATM inhibition may not be a suitable strategy to target cysts in ADPKD. However, further studies are needed to identify the hierarchical role of which DDR kinases (other than ATM) are essential for maintaining the increased survival of CECs. In this setting, the short-term treatment with a combination of a sensitizing agent together with the suppression of the DDR kinases that mediate CEC survival, may allow the acute and selective elimination of kidney cysts and allow the determination of this strategy on long-term disease progression in ADPKD.

## 4. Materials and Methods

### 4.1. Experimental Model of ADPKD and Method of ATM Inhibition

For genetic inhibition of ATM, mice with a truncating mutation in the ATM gene (Atmtm1Awb B6.129S6J; The Jackson Laboratory; #008536; Bar Harbor, ME, USA) [35] were crossed with C57BL/6J mice having knock-in of a PKD1 hypomorphic mutation (PKD1 p.R3277C) [34], to generate *Pkd1^RC/RC^/Atm^+/−^* mice as described previously [8]. Mouse colonies were maintained at the Australian BioResources (Moss Vale, NSW, Australia) and for experimental studies, transferred and housed at the Westmead Bioresources Facility, Westmead Institute for Medical Research (Westmead, NSW, Australia). Mice were housed under standard conditions (temperature: 21 ± 2 °C; humidity: 55 ± 15%; artificial lighting; light: dark cycle 1900–0700) and food and water were provided ad libitum. For pharmacological inhibition of ATM, the kinase inhibitor AZD0156 (Selleck Chemicals; Houston, TX, USA) was administered, as described below.

### 4.2. Experimental Design of the In Vivo Studies

Two experiments were performed in the *Pkd1^RC/RC^/Atm^+/−^* mice to determine the acute and chronic effects of cisplatin on the kidneys of the *Pkd1^RC/RC^/Atm^+/−^* mice. Heterozygous *Atm* mutation was used as the phenotype of mice homozygous for mutation is abnormal and exhibits growth retardation, neurological defects, and defects in immune cell function, and would confound results in the current study [35].In the first experiment to test the acute effect of single low-dose cisplatin, a range of doses of cisplatin (0.1, 0.5, 1.0, 3.5 or 7 mg/kg) or vehicle (normal saline) was injected intraperitoneally (i.p.) to *Pkd1^RC/RC^/Atm^+/−^* mice and 72 h post injection, kidneys were collected to observe tubular injury, DNA damage and apoptosis (n = 1 mouse per dose). In a subsequent experiment to test the effect of cisplatin on chronic inflammation and fibrosis [15], mice were injected i.p. cisplatin (1.0 or 7.0 mg/kg) or vehicle and kidneys were collected at 3-weeks post-injection to observe cyst formation, inflammation, and fibrosis (n = 5 *Pkd1^RC/RC^/Atm^+/−^* per group; n = 1 and n = 5 *Pkd1^+/+^/Atm^+/−^* in vehicle and cisplatin 7.0 mg/kg group, respectively, and n = 1 wildtype (*Pkd1^+/+^/Atm^+/+^*) in cisplatin 7.0 mg/kg and vehicle groups).

In the final experiment to test the acute effect of pharmacological inhibition of kinase activity of ATM, *Pkd1^RC/RC^* mice were given 20 mg/kg AZD0156, a dose that causes complete reduction of chemoresistance from ATM activity [38] and did not have toxic effects in *Pkd1^RC/RC^* mice [8]. AZD0156 (20 mg/kg) (n = 5) or vehicle (n = 4) was administered by oral gavage 1 day prior to i.p. cisplatin (7 mg/kg) and daily up to 72 h post cisplatin injection and mice were euthanized four hours following the final dose of AZD0156.

Kidney tissue was collected as previously described [8]. Briefly, mice were euthanized by an i.p. injection of ketamine:xylazine (100:10 mg/kg), and coronal slices from each kidney were snap-frozen or fixed in either 10% neutral-buffered formalin or methyl Carnoy’s solution and embedded in paraffin.

### 4.3. Histology and Immunohistochemistry

Histology and immunohistochemistry (IHC) were performed as previously described [8]. Briefly, kidney tissue sections (4 µm thick) fixed in methyl Carnoy’s solution were stained with periodic acid Schiff (PAS) stain and Sirus Red/Fast Green as previously described [6]. Primary antibodies used in IHC were γH2AX (Cell Signaling Technology, Danvers, MA, USA; 1:480), Cleaved Caspase-3 (Cell Signaling Technology, Danvers, MA, USA, 1:400), αSMA (Sigma-Aldrich, St Louis MO, USA; 1:4000), F4/80 (abD Serotec, 1:300). Biotin-conjugated secondary antibodies (1:200) and vectastain ABC reagent (Vector Laboratories, Burlingame, CA, USA) were used, followed by diaminobenzidine (DAB). Sections were counterstained with methyl green. Images were obtained using a slide scanner (NanoZoomer v1; Hamamatsu Photonics, Iwata, Japan), and quantified using the positive pixel algorithm on Aperio ImageScope version 11.2.0.780 (Leica Biosystems, Wetzlar, Germany). For percentage positive pixels and pixel intensity, a total of 5–10 random 10× fields of view from the kidney cortex were analyzed separately for cyst lining cells and non-cystic tissue and averaged for each sample.

### 4.4. Semi-Quantitative Assessment of Tubular Injury

Tubular injury in mice three weeks post cisplatin injection were scored semi-quantitatively in PAS-stained kidney sections. Images of five to ten fields of view (20× objective) per section were captured starting at the 12 o’clock position and moving clockwise at equally spaced distances. For quantification, each of the 20× fields of view was divided into quarters and scored 0, 0.5 or 1.0 per quarter depending on extent of injury (tubular dilation, cystic dilation, sloughing of epithelial cells, interstitial widening, casts, basement membrane thickening and inflammatory infiltrates). The average score per mouse was calculated from the 5–10 fields of view per section.

### 4.5. ADPKD and Kidney Cell Lines

Human ADPKD cells WT9-7 and WT9-12 [39], and control HK-2 cells [40] were obtained from the American Type Culture Collection (ATCC; Manassas, VA; CRL-2830, lot number 58737172; CRL-2833, lot number 60336584 and CRL-2190, lot number 61218770, respectively). The Madin-Darby canine kidney (MDCK) Type I epithelial cells used in the three-dimensional cyst growth assay were obtained from European Collection of Authenticated Cell Cultures (ECACC; 00062106). All cell lines were maintained in culture at 37 °C in 5% CO_2_ in the presence of 10% fetal bovine serum enriched media as previously described [6].

### 4.6. Assessment of Cell Survival

Cells were treated for two hours in the presence of cisplatin (0.5, 1.0, 2.0, 4.0, 8.0 µg/mL) or vehicle and the media was replenished to allow recovery for 72 h before cell viability determination using the MTT assay (Cell Proliferation Kit I, 11465007001; Roche) according to manufacturer’s instructions. Absorbance was measured at 570 nm with 750 nm as reference wavelength. Technical replicates included four individual wells per group.

### 4.7. Three-Dimensional Model of In Vitro Cyst Growth Assay

Three-dimensional (3D) MDCK cysts were cultured in collagen matrix support as previously described [8]. The effect of cisplatin (0.25 and 1.0 µg/mL) on established cysts (Day 4 since plating) was tested by exposure for 2 h followed by recovery through day 12. Forskolin enriched media was replenished every two days and cyst images were captured at a fixed field (center of well) and depth of view using a 5× objective at days 4 (baseline), 8 and 12. Cysts in focus at the specified depth were selected by starting at the top left of the field and proceeding by rows from left to right (n = 8–10 cysts/well and 3 wells/treatment). Cyst diameter was measured from captured images using Image J (version 1.52a).

### 4.8. Statistical Analysis

Numerical data were analyzed using Excel, JMP Pro version 14.2.0 (SAS Institute, Cary, NC, USA) and GraphPad. Data are presented as means ± SD or median ± interquartile range. Two-sample independent t-test or one-way analysis of variance followed by post hoc Tukey–Kramer honestly significant difference (HSD) test was used for testing difference between normally distributed groups and Wilcoxon test for non-parametric data. *p* values< 0.05 were considered statistically significant.

## Supplementary Materials

The supporting information can be downloaded at: https://www.mdpi.com/article/10.3390/ijms232012547/s1.
