# Supplementary material for: Kidney Cyst Lining Epithelial Cells Are Resistant to Low-Dose Cisplatin-Induced DNA Damage in a Preclinical Model of Autosomal Dominant Polycystic Kidney Disease
Source: Int J Mol Sci. 2022 Oct 19;23(20):12547. doi: 10.3390/ijms232012547 (PMC9603998; doi:10.3390/ijms232012547)
Supplement: Supplementary file 1 [file ijms-23-12547-s001.zip › ijms-1880985-supplementary.pdf]

Figure S1. Representative images of PAS staining (upper panel) and immunostaining for γH2AX (bottom panel) of Pkd1RC/RC/Atm+/− mice kidneys showing dose response to cisplatin (0.1 mg/kg to 7.0 mg/kg) at 72 hours post injection. Scale bar = 50 μm.

Figure S2. Morphological staining (PAS) of Pkd1RC/RC/Atm+/− mice kidney sections at 3 weeks post cisplatin injections (1 mg/kg or 7 mg/kg) or vehicle. Scale bar = 1mm.

Figure S3. Immunohistochemical staining for F4/80 in Pkd1RC/RC/Atm+/− mice kidney sections at 3 weeks post cisplatin injections (1 mg/kg or 7 mg/kg) or vehicle (7 mg/kg). Scale bar = 1mm.

Figure S4. Sirius red staining indicating fibrosis (pink) in Pkd1RC/RC/Atm+/− mice kidney sections at 3 weeks post cisplatin injections (1 mg/kg or 7 mg/kg) or vehicle (7 mg/kg). Scale bar = 200 μm.

Figure S5. Immunostaining for αSMA in Pkd1RC/RC/Atm+/− mice kidney sections at 3 weeks post cisplatin injections (1 mg/kg or 7 mg/kg) or vehicle (7 mg/kg). Scale bar = 1mm.

Figure S1

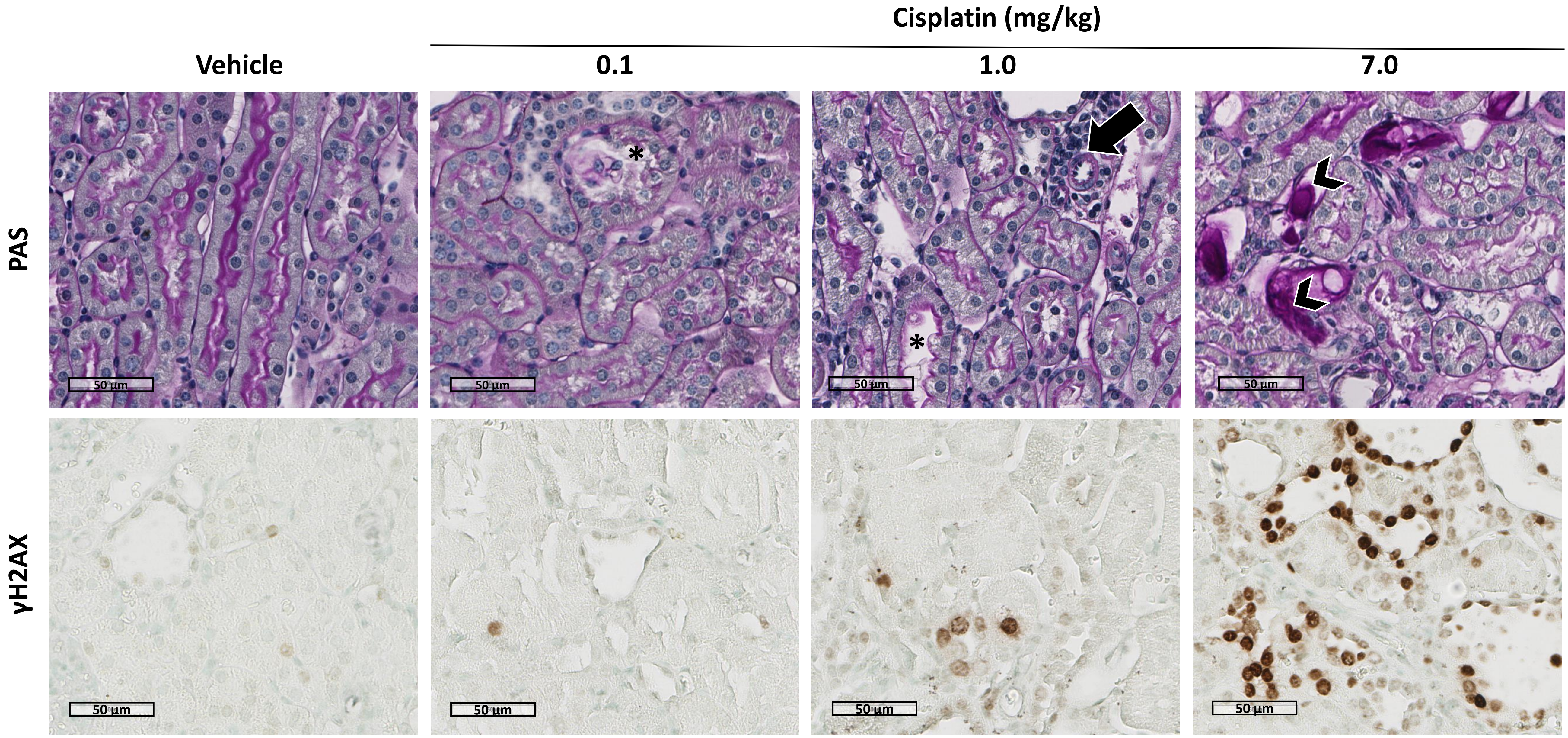

Figure S2

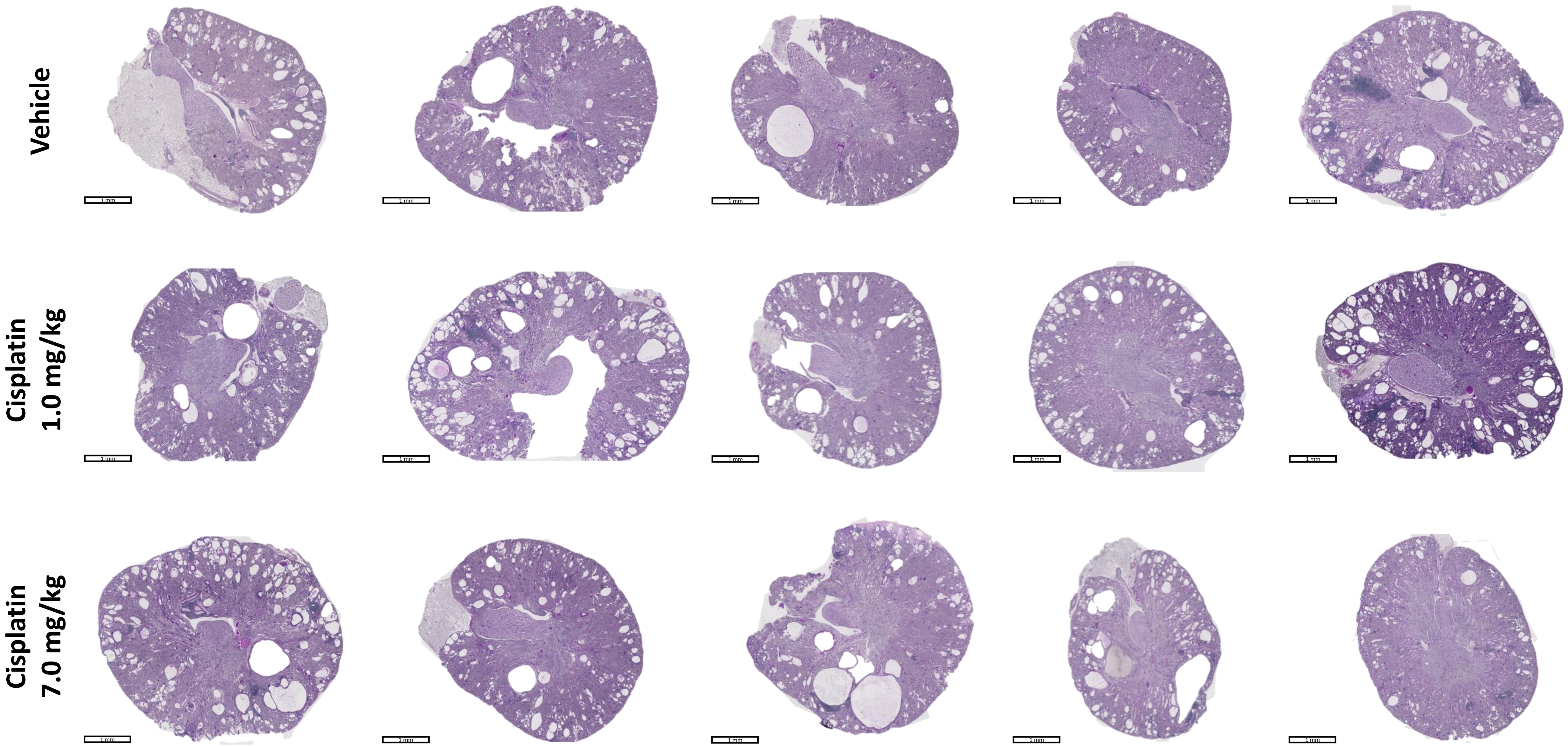

Figure S3

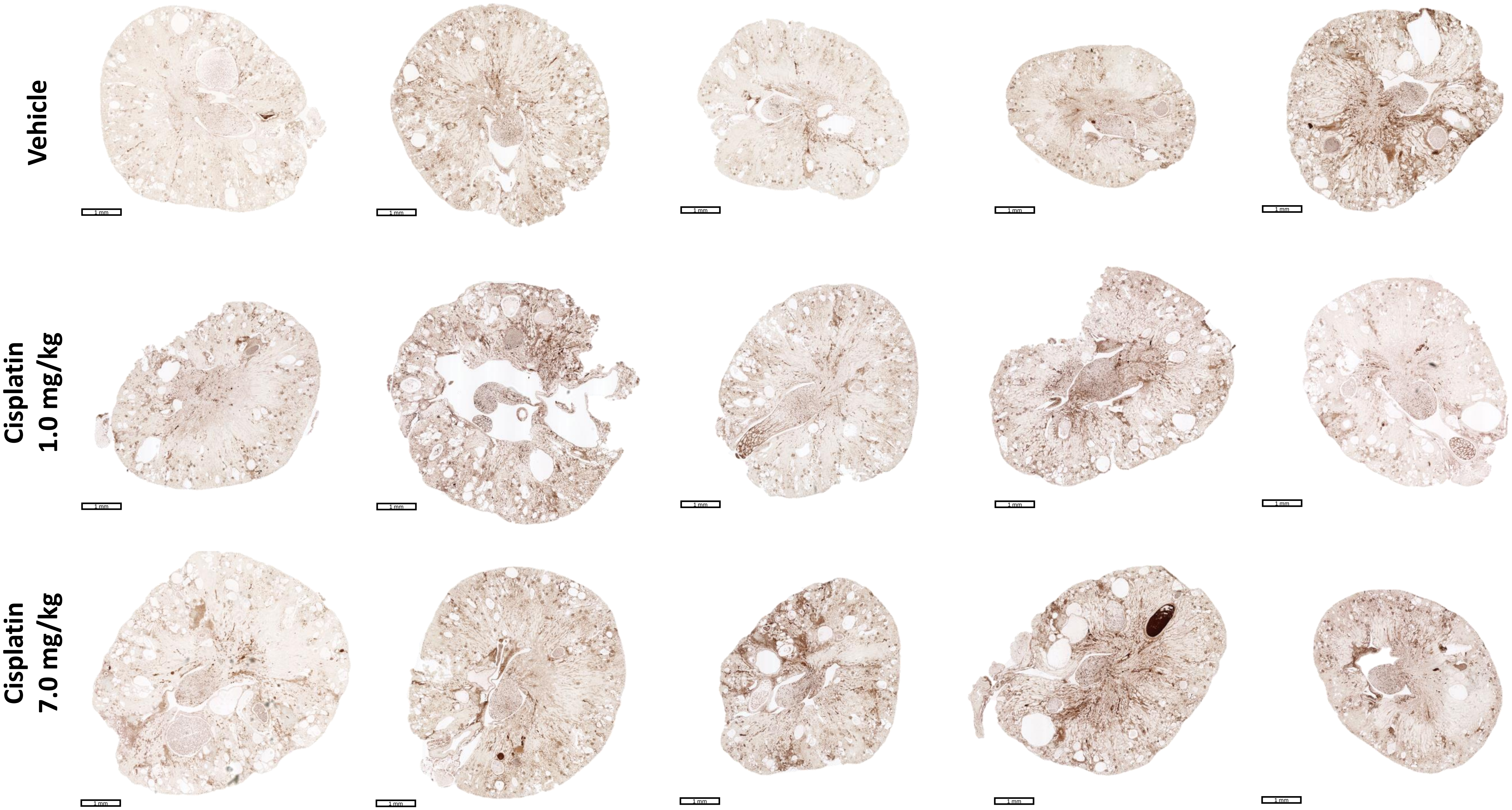

Figure S4

Vehicle	Cisplatin<br>1.0 mg/kg	Cisplatin<br>7.0 mg/kg

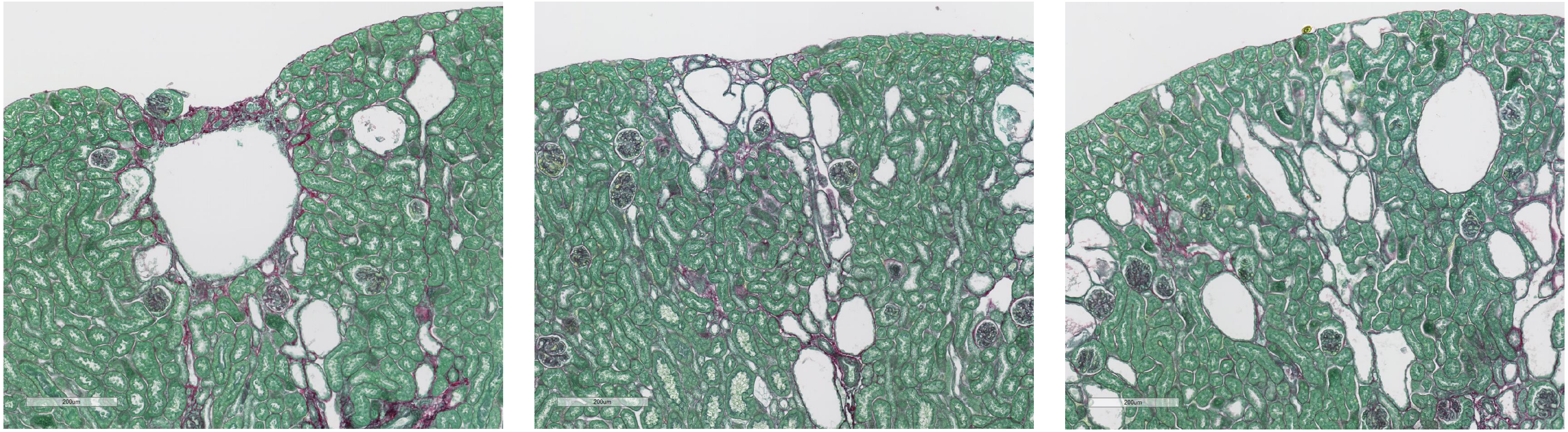

Figure S5

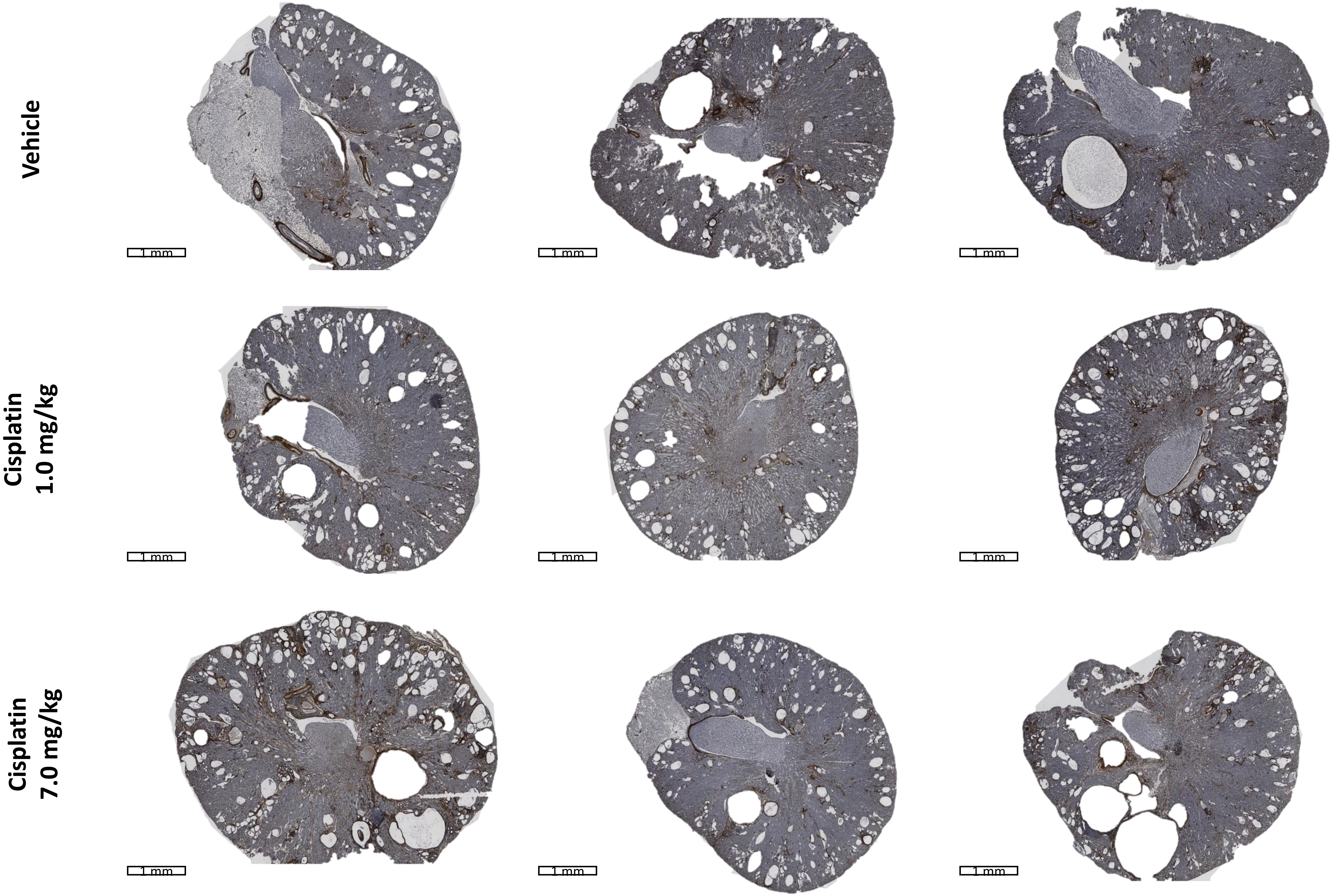
Vehicle
Cisplatin 1.0 mg/kg
Cisplatin 7.0 mg/kg
1 mm
1 mm
1 mm
1 mm
1 mm
1 mm
1 mm
1 mm
1 mm
